# Comparative conventional and phenomics approaches to assess symbiotic effectiveness of Bradyrhizobia strains in soybean (*Glycine max* L. Merrill) to drought

**DOI:** 10.1038/s41598-017-06441-3

**Published:** 2017-07-31

**Authors:** Venkadasamy Govindasamy, Priya George, Lalitkumar Aher, Shunmugiah V. Ramesh, Arunachalam Thangasamy, Sivalingam Anandan, Susheel Kumar Raina, Mahesh Kumar, Jagadish Rane, Kannepalli Annapurna, Paramjit Singh Minhas

**Affiliations:** 1 School of Drought Stress Management, ICAR-National Institute of Abiotic Stress Management, Baramati, Pune, 413115 Maharashtra India; 2 Division of Microbiology, ICAR-Indian Agricultural Research Institute, New Delhi, 110012 India; 3 ICAR-Indian Institute of Soybean Research, Indore, 452001 Madhya Pradesh India; 4 ICAR-Directorate of Onion and Garlic Research, Rajgurunagar, Pune, 410505 Maharashtra India; 5 ICAR-Central Institute of Temperate Horticulture, Srinagar, 190007 Jammu and Kashmir India

## Abstract

Symbiotic effectiveness of rhizobitoxine (Rtx)-producing strains of *Bradyrhizobium* spp. in soybean (cultivar NRC-37/Ahilya-4) under limited soil moisture conditions was evaluated using phenomics tools such as infrared(IR) thermal and visible imaging. Red, green and blue (RGB) colour pixels were standardized to analyse a total of 1017 IR thermal and 692 visible images. Plants inoculated with the Rtx-producing strains *B. elkanii* USDA-61 and USDA-94 and successive inoculation by *B. diazoefficiens* USDA-110 resulted in cooler canopy temperatures and increased canopy greenness. The results of the image analysis of plants inoculated with Rtx-producing strains were correlated with effective nodulation, improved photosynthesis, plant nitrogen status and yield parameters. Principal component analysis (PCA) revealed the reliability of the phenomics approach over conventional destructive approaches in assessing the symbiotic effectiveness of *Bradyrhizobium* strains in soybean plants under watered (87.41–89.96%) and water-stressed (90.54–94.21%) conditions. Multivariate cluster analysis (MCA) revealed two distinct clusters denoting effective (Rtx) and ineffective (non-Rtx) *Bradyrhizobium* inoculation treatments in soybean. Furthermore, correlation analysis showed that this phenotyping approach is a dependable alternative for screening drought tolerant genotypes or drought resilience symbiosis. This is the first report on the application of non-invasive phenomics techniques, particularly RGB-based image analysis, in assessing plant-microbe symbiotic interactions to impart abiotic stress tolerance.

## Introduction

The occurrence and magnitude of abiotic stresses might increase in the near future because of global climate change. Severe abiotic stress due to frequent drought and other edaphic factors is evident particularly in crop production systems^1^. To mitigate the effects of these stresses and to increase crop productivity, appropriate crop management techniques are imperative. Several management system techniques, such as crop rotation, intercropping, row skipping (decreasing planting density by omitting rows), mulching, protected cropping (crops grown under glass, plastic or nets) and biofertilization, could be employed to improve crop productivity^2^. Breeding for high crop yields in abiotic stress-prone environments is complicated due to other abiotic factors such as the temporal distribution of available soil water and the low heritability of tolerance traits under these conditions^3^. The use of poor or inadequate low cost phenotyping methodologies remains an important constraint that hinders the development and adoption of improved technologies pertaining to plant stress management^3^.

Among the abiotic factors that drive plant evolution and crop production, water availability is of prime importance^4^. Moisture stress affects virtually every aspect of plant physiology and metabolism. Although some of the physiological and metabolic changes observed under these stresses are adaptive, many are the consequences of stress injury^5^. Low soil moisture stress adversely affects the oxygen diffusion barrier that is crucial for the effective functioning of legume nodules^6^. It has also been reported that symbiotic nitrogen fixation (SNF) is rapidly inhibited by water deprivation, as the deprivation causes changes in nodule morphology and metabolism^7^. The legume/*Rhizobium* symbiosis is destabilized by drought or low soil moisture, causing poor nodulation and premature nodule senescence^8, 9^. These findings have unequivocally demonstrated that SNF is highly sensitive to water availability in the root environment.

*Rhizobium* spp. often fail to nodulate the plant when they are exposed to stress conditions such as wounding, flooding, chemicals or heavy metals, low soil moisture or drought, soil salinity, extreme temperatures, and pathogen infection. Under stress conditions, ethylene, regarded as a stress hormone, is produced and inhibits overall plant growth, which includes legume-*Rhizobium* symbiosis^10^. It was suggested that rhizobia employ at least two strategies to reduce the amount of host legume-derived ethylene to counteract the negative effect of ethylene on nodulation^11^. The production of rhizobitoxine [2-amino-4-(2-amino-3-hydropropoxy)-*trans*-but-3-enoic acid] by slow-growing *Bradyrhizobium* spp. and ACC deaminase by fast-growing *Rhizobium* spp. has been observed. Rhizobitoxine (Rtx) competitively inhibits 1-aminocyclopropane-1-carboxylate (ACC) synthase, a key enzyme of the ethylene biosynthesis pathway in both tomato^12^ and *Macroptilium atropurpureum* ^13^. The beneficial effects of the inoculation of rhizobitoxine-producing strains and improved nodulation in fertile fields and under irrigated conditions have been reported^11, 13, 14^. Rtx-producing rhizobia can increase nodulation but can also reduce the growth of sirato plants by accumulating more storage lipids and gaining an advantage^15^. However, the Rtx-producing traits of bradyrhizobial strains on symbiotic effectiveness and plant phenotypic responses are poorly understood under abiotic stresses, particularly in nutrient-poor soils and low soil moisture stress conditions.

Soybean (*Glycine max* (L.) Merrill) is a globally important commercial crop that is grown mainly for its protein, oil and nutraceutical contents^16, 17^. Soybean establishes symbiotic interactions with N_2_-fixing bacteria that belong to the rhizobia group, specifically *Bradyrhizobium* spp. (slow growers) and *Ensifer* spp. (formerly called *Sinorhizobium*, fast growers)^18^. The beneficial effects of *Bradyrhizobium* spp. on the growth and yield parameters of soybean and other leguminous hosts under irrigated conditions based on conventional approaches are well documented^19–23^. However, the understanding and assessment of plant responses to these symbiotic interactions on both plant physiology (plant canopy features, plant water status and photosynthetic efficiency) and biochemical metabolism and their impact due to improved root nodulation and nitrogen fixation through advanced phenomics approaches under low soil moisture stress conditions/moderate drought stress are poor^24^.

In the present investigation, we used a soybean-*Bradyrhizobium* symbiotic system as a model to investigate plant physiological responses (plant canopy features and health of photosynthetic systems) upon inoculation with rhizobitoxine (Rtx)-producing bradyrhizobial strains under optimum and low soil moisture or imposed moderate drought stress conditions. We hypothesized that phenotyping/phenomics techniques such as red, green and blue (RGB)-based image analysis of aerial plant parts possibly could provide deeper insights into plant physiology and metabolic responses to drought stress during symbiotic interactions. It also helps to differentiate the effectiveness of symbiotic interactions upon inoculation with bradyrhizobial strains in soybean^24^. We applied a visible and infrared thermal imaging-based phenomics approach along with non-destructive photosynthetic measurements to assess the symbiotic effectiveness of non-Rtx- and Rtx-producing strains of *Bradyrhizobium* spp. in soybean under optimum and low soil moisture conditions. Furthermore, the superiority of this image-based phenomics approach over conventional approaches of assessing nodulation, plant nitrogen status, plant growth, yield parameters and rhizosphere microbiological properties during soybean-*Bradyrhizobium* symbiotic interactions under limited soil moisture or imposed drought stress was validated.

## Results

### Screening of Bradyrhizobial strains for the rhizobitoxine production trait

PCR screening of bradyrhizobial strains (using the primer set *rtxA* F and *rtxA* R) revealed the presence of the *rtxA* gene with expected amplicons of ~2500 bp in size for rhizobitoxine-producing strains *B. elkanii* USDA-61 and 94; in contrast, *B. diazoefficiens* USDA-110 did not show any amplification (Supplementary Fig. S1). However, the PCR amplification of a partial *rtxA* gene showed expected amplicons of 1250 bp in two rhizobitoxine-producing strains(*B. elkanii* USDA-61 and USDA-94) and from *B. diazoefficiens* strain USDA-110 (Supplementary Fig. S1). Later, the amplicons were sequenced and confirmed by BLASTn analysis. Hence, a second pair of degenerate primers was used for screening all 86 bradyrhizobial isolates for the presence of the partial *rtxA* gene using the genomic DNA of *B. diazoefficiens* strain USDA-110 as a positive reference. However, none of the 83 bradyrhizobial strains showed PCR amplification, confirming the absence of the *rtxA* gene (Supplementary Fig. S1).

### Standardization of RGB software, visible and IR imaging of the plant canopy and image analysis vs root nodulation

**Figure 1:**
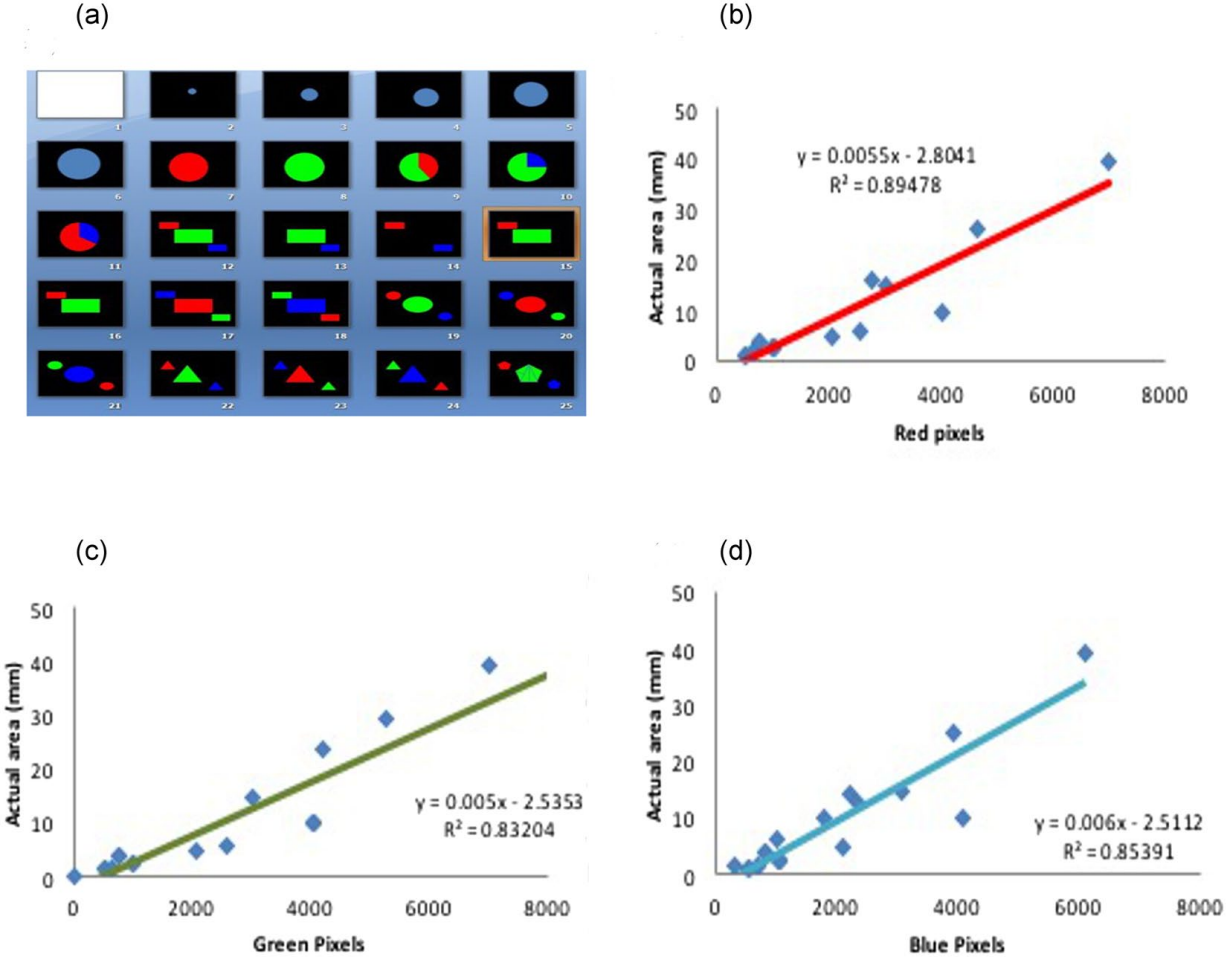
Combined figure showing schemes of primary colours (red, green and blue) and their combinations/mixtures (**a**) used for the standardization of image analysis software. Standard curves representing actual colour area *vs* number of colour pixels for red (**b**), green (**c**) and blue (**d**) after the standardization of RGB image analysis software.

**Figure 2:**
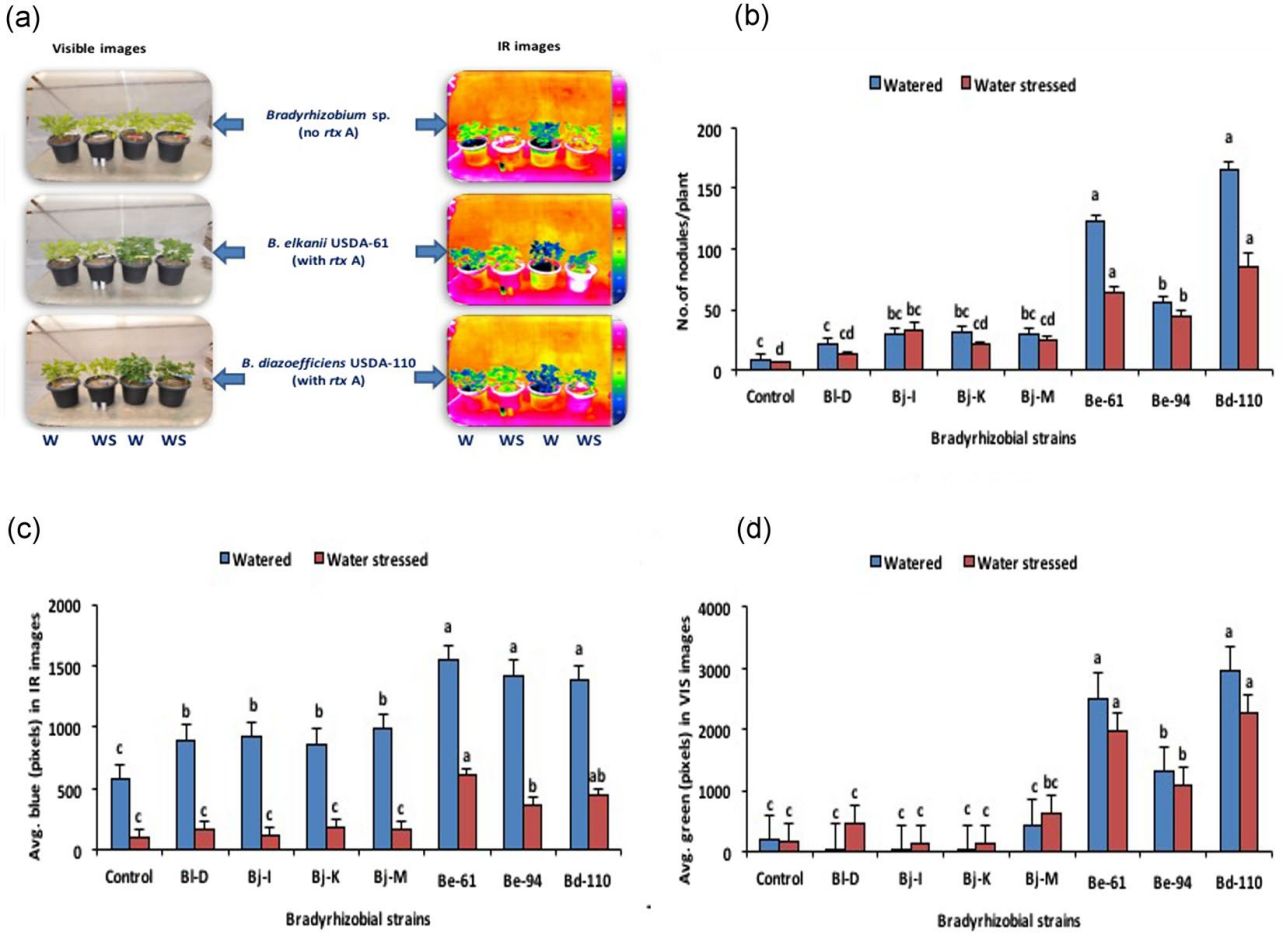
Comparative visible and IR thermal images showing plant canopy features of soybean plants inoculated and not inoculated with Rtx and non-Rtx bradyrhizobial strains (**a**) under watered and water-stressed conditions. Effect of the inoculation of Rtx and non-Rtx bradyrhizobial strains on root nodulation (**b**) and canopy measurements in IR thermal images (**c**) and visible images (**d**) of inoculated soybean plants under watered and water-stressed conditions.

The software for RGB-based image analysis was standardized with the images of defined resolution (0–10000 pixels) for the primary colours red, green and blue and their combinations (Fig. 1a). Standard curves delineating the relationship between the actual area in the red image and its corresponding known red pixels were drawn (Y = 0.005x − 2.804; R^2^ = 0.894) to directly measure red pixels of any unknown plant image. Similarly, the standard curves depicting the relationships between the actual area and pixels for green (Y = 0.005x − 2.535; R^2^ = 0.832) and blue (Y = 0.005x − 2.511; R^2^ = 0.853) images were also drawn to measure their respective colour pixels from the plant images (Fig. 1b–d). Canopy images of watered soybean plants usually showed more green pixels in visible and blue pixels in IR thermal images compared to the images of water-stressed plants (Fig. 2a). These observed variations in the plant canopy features could be due to differences in the nodulation effectiveness of inoculated bradyrhizobia (Fig. 2b). In general, soybean nodulation was found to be reduced in both non-Rtx- and Rtx-producing bradyrhizobial strains upon inoculation and control plants grown under water-stressed conditions compared to regularly watered plants. The maximum number of nodules among the plants under low moisture stress conditions was found in the plants inoculated with *B. diazoefficiens* USDA-110 (85.00 ± 12.26) and successive inoculation with Rtx-producing strain *B. elkanii* USDA-61 (64.50 ± 3.68); these numbers of nodules were significantly higher than those of plants inoculated with *B. elkanii* USDA-94 (44.66 ± 3.82). However, the number of nodules of soybean plants was much higher upon inoculation with *B. diazoefficiens* USDA-110 (166.17 ± 13.67), *B. elkanii* USDA-61 (122.83 ± 9.80) and *B. elkanii* USDA-94 (55.33 ± 4.90) under well-watered conditions(Fig. 2b). Among the non-Rtx-producing strains, plants inoculated with *B. japonicum* strain Bj-I showed increased nodulation under water-stressed conditions, which was statistically equal to that of treatments inoculated with other non-Rtx strains. To further correlate the effectiveness of nodulation and plant canopy features, image analysis was performed. Using the image analysis software, a total of 1017 IR thermal images were analysed for their red, green and blue pixels, which represented high, moderate and low canopy temperatures respectively. Around 692 visible images were analyzed for green pixels which indicated canopy greenness. The image analysis revealed that plants inoculated with Rtx-producing strains of *B. elkanii* USDA-61 showed high amounts of blue pixels (~1600 pixels), indicating the coolest canopy, which was on par with inoculation of *B. diazoefficiens* strain USDA-110 and *B. elkanii* strain USDA-94 compared to plants inoculated with non-Rtx strains and non-inoculated plants under water-stressed conditions (Fig. 2c). From the analysis of visible-range images, it was observed that the average number of green pixels was significantly higher in soybean inoculated with the strain of *B. diazoefficiens* USDA-110 (~3000 pixels) (Fig. 2d). This result was on par with that of the Rtx-producing strains *B. elkanii* USDA-61 and successive inoculation by USDA-94, indicating higher canopy greenness compared to both plants inoculated with other non-Rtx strains and uninoculated soybean plants under water-stressed conditions (Fig. 2d).

### Relative chlorophyll content and quantum efficiency of photo system II

**Figure 3:**
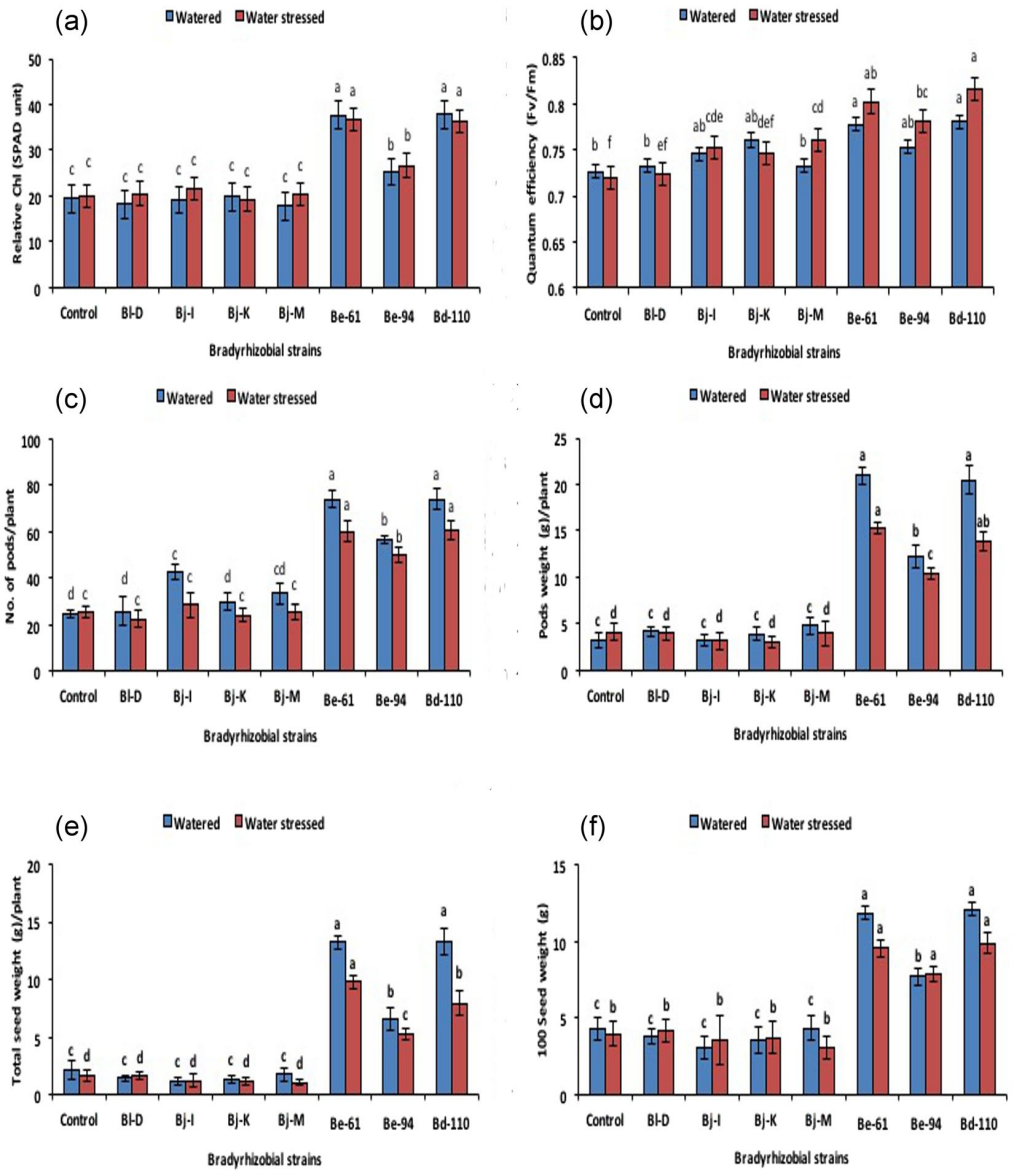
Inoculation effect of Rtx and non-Rtx bradyrhizobial strains on SPAD relative chlorophyll content (**a**), the quantum efficiency of PSII (**b**), number of pods (**c**), pot weight (**d**), total seed yield (**e**) and test seed weight (**f**) in soybean plants under watered and water-stressed conditions.

Relative chlorophyll content levels were higher in watered soybean plants compared to those in plants grown under water stress. Plants inoculated with *B. diazoefficiens* strain USDA-110 and the Rtx-producing strain *B. elkanii* USDA-61 showed the highest relative chlorophyll content in terms of SPAD units (>35). The highest was followed by *B. elkanii* USDA-94 compared to plants that were inoculated with other non-Rtx strains and uninoculated plants under water-stressed conditions (Fig. 3a). Photosynthetic efficiency measurements in soybean revealed that plants inoculated with Rtx-producing strains showed higher quantum efficiency of PSII (*F*v/*F*m) compared to plants inoculated with other strains under both water-stressed and regularly watered conditions (Fig. 3b). Plants inoculated with *B. diazoefficiens* strain USDA-110 (*F*v/*F*m = 0.82) and the Rtx-producing strain *B. elkanii* USDA-61 (*F*v/*F*m = 0.80) revealed significantly higher quantum efficiency of PSII and successive inoculation by *B. elkanii* USDA-94 compared to plants inoculated with other non-Rtx strains and uninoculated controls under water-stressed conditions.

### Chlorophyll content of soybean plants inoculated with bradyrhizobial strains

To validate the results obtained from image analysis with those of relative chlorophyll content obtained through SPAD measurements, wet lab chlorophyll estimation was carried out (Table 1). In general, inoculation with *B. diazoefficiens* strain USDA-110 [water-stressed (WS)- 3.109 ± 0.34, 0.676 ± 0.04 and 3.785 ± 0.38 mg/g leaf FW; watered (W)- 3.042 ± 0.35, 0.613 ± 0.09 and 3.655 ± 0.44 mg/g leaf FW) and the Rtx-producing strain *B. elkanii* USDA-61 (WS- 2.684 ± 0.01, 0.574 ± 0.0 and 3.257 ± 0.01 mg/g leaf FW; W- 2.388 ± 0.20, 0.477 ± 0.05 and 2.864 ± 0.24 mg/g leaf FW)] resulted in significant increases in the leaf chlorophyll a, b, and a + b compared to plants inoculated with non-Rtx strains and uninoculated control treatments, under both water-stressed and regularly watered conditions. *Bradyrhizobium diazoefficiens* strain USDA-110-inoculated treatments were found to show significant increases in leaf chlorophyll a, b, and a + b followed by two rhizobitoxine-producing *B. elkanii* strains compared to other bradyrhizobial strains. Among the non-Rtx-producing strains, plants inoculated with *B. japonicum* strain Bj-I showed increases in leaf chlorophyll a, b, and a + b under watered conditions, whereas *B. japonicum* strain Bj-K displayed increased leaf chlorophyll under water-stressed conditions.

**Table 1:** Inoculation effects of non-rhizobitoxine- and rhizobitoxine-producing *Bradyrhizobium* strains on the chlorophyll content of soybean plants using the DMSO method under watered and water-stressed conditions.

Treatments	Watered^†^			Water stressed^†^		
	Chl ‘a’ (mg/g leaf FW)	Chl ‘b’ (mg/g leaf FW)	Chl ‘a + b’ (mg/g leaf FW)	Chl ‘a’ (mg/g leaf FW)	Chl ‘b’ (mg/g leaf FW)	Chl ‘a + b’ (mg/g leaf FW)
Control	1.040 ± 0.11^c^	0.231 ± 0.02^cd^	1.271 ± 0.13^c^	0.762 ± 0.12^c^	0.187 ± 0.03^c^	0.949 ± 0.15^c^
*B. liaoningense* strain D (Bl-D)	1.061 ± 0.01^c^	0.231 ± 0.00^cd^	1.292 ± 0.01^c^	0.667 ± 0.02^c^	0.152 ± 0.00^c^	0.819 ± 0.02^c^
*B. japonicum* strain I (Bj-D)	1.932 ± 0.12^b^	0.372 ± 0.02^bc^	2.303 ± 0.14^b^	0.817 ± 0.07^c^	0.197 ± 0.01^c^	1.014 ± 0.08^c^
*B. japonicum* strain K (Bj-K)	0.889 ± 0.19^c^	0.197 ± 0.03^d^	1.085 ± 0.22^c^	0.881 ± 0.06^c^	0.199 ± 0.01^c^	1.080 ± 0.06^c^
*B. japonicum* strain M (Bj-M)	1.657 ± 0.13^bc^	0.344 ± 0.01^bcd^	2.001 ± 0.14^bc^	0.854 ± 0.02^c^	0.224 ± 0.02^c^	1.078 ± 0.03^c^
*B. elkanii* strain USDA-61 (Be-61)	2.388 ± 0.20^a^	0.477 ± 0.05^a^	2.864 ± 0.24^a^	2.684 ± 0.01^ab^	0.574 ± 0.00^ab^	3.257 ± 0.01^ab^
*B. elkanii* strain USDA-94 (Be-94)	2.869 ± 0.04^ab^	0.546 ± 0.02^ab^	3.414 ± 0.06^ab^	2.189 ± 0.10^b^	0.443 ± 0.01^b^	2.631 ± 0.12^b^
*B. diazoefficiens* strain USDA-110 (Bd-110)	3.042 ± 0.35^a^	0.613 ± 0.09^a^	3.655 ± 0.44^a^	3.109 ± 0.34^a^	0.676 ± 0.04^a^	3.785 ± 0.38^a^

^†^Values are the means of six-replication ± standard error. Values with the same superscripts within column indicate no significant difference with *P* ≥ 0.05.

### Improvement in plant growth and yield parameters

Plant growth and biomass accumulation, i.e., shoot and root length, the number of branches, and the dry weight of shoots and roots, were reduced when soybean was grown under water-stressed conditions compared to watered conditions (Table 2). Plants inoculated with B. diazoefficiens strain USDA-110 and Rtx-producing strains, i.e., *B. elkanii* USDA-61 and *B. elkanii* USDA-94, showed the highest shoot and root length, number of branches, and shoot and root dry weight compared to plants inoculated with non-Rtx strains and control treatments in both water-stressed and watered conditions. The number of pods, pod and seed weight per plant and seed test weight (100 seeds weight) under both watered and water-stressed conditions showed increases in soybean plants inoculated with Rtx-producing bradyrhizobial strains compared to plants inoculated with non-Rtx strains (Fig. 3c–f). Under water-stressed conditions, soybean inoculated with *B. diazoefficiens* strain USDA-110 showed a significant increase in the number of pods (60.75 ± 4.11), followed by inoculation with Rtx-producing strains *B. elkanii* USDA-61 (60.07 ± 4.47) and *B. elkanii* USDA-94 (49.77 ± 3.16) (Fig. 3c). However, the pod weight of soybean inoculated with *B. elkanii* USDA-61 (15.20 ± 0.62) and *B. diazoefficiens* USDA-110 (13.87 ± 0.96) was significantly higher than that of *B. elkanii* USDA-94 (10.33 ± 0.65) (Fig. 3d). Thus, inoculation with all three strains significantly improved the pod weight of soybean compared to inoculation with both non-Rtx strains and uninoculated control treatments under low soil moisture stress conditions.

**Table 2:** Inoculation effects of non-rhizobitoxine- and rhizobitoxine-producing *Bradyrhizobium* strains on plant growth and biomass accumulation of soybean plants under watered and water-stressed conditions.

Treatment	Watered^†^					Water stressed^†^				
	Length (cm)		No of Branches	Dry weight (g)		Length (cm)		No of Branches	Dry weight (g)	
	Shoot	Root		Shoot	Root	Shoot	Root		Shoot	Root
Control	38.28 ± 2.21^c^	48.48 ± 4.16^c^	5.97 ± 0.32^d^	26.08 ± 1.60^c^	16.18 ± 0.52^c^	38.69 ± 2.04^b^	47.91 ± 6.31^b^	6.66 ± 0.49^d^	18.87 ± 2.25^b^	10.56 ± 0.40^c^
*B. liaoningense* strain D (Bl-D)	41.97 ± 1.10^b^	53.38 ± 3.17 ^b^	6.50 ± 0.41^c^	28.60 ± 3.41^bc^	16.84 ± 0.65^bc^	40.06 ± 1.83^b^	51.18 ± 4.78^b^	6.94 ± 0.40^c^	19.33 ± 0.49^b^	11.22 ± 1.03^b^
*B. japonicum* strain I (Bj-D)	40.94 ± 2.18^b^	54.39 ± 3.74^b^	7.00 ± 0.71^b^	30.85 ± 2.58^b^	17.71 ± 1.26^bc^	40.83 ± 1.82^b^	51.61 ± 4.43^b^	7.11 ± 0.47^b^	19.71 ± 1.93^b^	11.41 ± 0.48^b^
*B. japonicum* strain K (Bj-K)	40.35 ± 0.42^b^	52.78 ± 2.39^b^	6.47 ± 0.10^c^	28.67 ± 2.17^bc^	16.59 ± 1.19^bc^	39.39 ± 0.85^b^	50.66 ± 3.46^b^	6.85 ± 0.06^c^	18.66 ± 0.37^b^	10.85 ± 1.36^b^
*B. japonicum* strain M (Bj-M)	41.91 ± 1.18^b^	53.44 ± 4.65^b^	6.40 ± 0.15^c^	29.50 ± 3.53^bc^	16.76 ± 0.55^bc^	41.32 ± 1.27^b^	50.24 ± 2.05^b^	7.05 ± 0.20^c^	19.57 ± 3.10^b^	11.35 ± 1.42^b^
*B. elkanii* strain USDA-61 (Be-61)	48.27 ± 1.90^a^	66.38 ± 1.66^a^	8.94 ± 0.28^a^	33.94 ± 2.71^a^	18.61 ± 0.37^a^	46.05 ± 2.36^a^	56.66 ± 0.77^a^	8.27 ± 0.62^a^	22.77 ± 3.36^a^	12.98 ± 1.09^a^
*B. elkanii* strain USDA-94 (Be-94)	46.76 ± 0.61^a^	62.11 ± 3.53^a^	8.55 ± 0.29^a^	32.59 ± 2.28^a^	18.30 ± 0.78^a^	43.08 ± 0.72^a^	57.28 ± 5.42^a^	8.19 ± 0.44^a^	22.65 ± 4.19^a^	12.62 ± 0.99^a^
*B. diazoefficiens* strain USDA-110 (Bd-110)	48.64 ± 0.86^a^	67.23 ± 2.12^a^	8.78 ± 0.19^a^	35.83 ± 1.71^a^	20.43 ± 1.01^a^	46.61 ± 1.35^a^	55.39 ± 1.99^a^	8.94 ± 0.70^a^	29.92 ± 3.12^a^	14.08 ± 1.12^a^

^†^Values are the means of six-replication ± standard error. Values with the same superscripts within column indicate no significant difference at *P* ≥ 0.05.

The inoculation of soybean with Rtx-producing *B. elkanii* strains and *B. diazoefficiens* USDA-110 significantly increased total seed weight to 13.28 ± 1.2 g/plant and 7.9 ± 1.1 g/plant under watered and water-stressed conditions, respectively, compared to uninoculated control treatments (2.5 ± 0.74 and 1.64 ± 0.49 g/plant) (Fig. 3e). Seed test weight (100-seed weight) of the soybean plants significantly increased after inoculation with *B. diazoefficiens* USDA-110 (12.1 ± 0.47 g) and *B. elkanii* USDA-61 (11.86 ± 0.38 g) under watered conditions and with *B. diazoefficiens* USDA-110 (9.9 ± 0.71 g) and *B. elkanii* USDA-61 (9.6 ± 0.55 g) under water-stressed conditions (Fig. 3f). A significant improvement in test seed weight was observed in soybean inoculated with Rtx-producing strains compared to plants inoculated with non-Rtx strains and uninoculated control treatments imposed low soil moisture stress conditions.

### Improvement in available nitrogen of rhizosphere soil and plant N status of soybean

Indirectly, the inoculation of bradyrhizobial strains improved the available nitrogen content in soybean rhizosphere soil, showing an increase of 85.72 ± 3.86 kg/ha (Bj-I and Bj-M strain inoculations) – 114 ± 3.86 kg/ha (Bd-110 inoculation) compared to uninoculated controls (83.63 kg/ha) under watered conditions (Table 3). However, the inoculation of these strains increased the soil available nitrogen content in the range of 77.35 ± 2.09 kg/ha (Bl-D inoculation) – 108.71 ± 4.18 kg/ha (Bj-110 inoculation) compared to uninoculated controls under water-stressed conditions. Under both watered and water-stressed conditions, the inoculation of Rtx-producing bradyrhizobial strains showed tremendous increases in the available nitrogen of the soybean rhizosphere compared to those of the inoculation of non-Rtx-producing strains, uninoculated controls and absolute controls[the pre-sown soil (49.93 ± 2.67 kg/ha)].

**Table 3:** Inoculation effects of non-rhizobitoxine- and rhizobitoxine-producing *Bradyrhizobium* strains on the available nitrogen in the rhizosphere and total nitrogen content of soybean plants under watered and water-stressed conditions.

Treatment	Watered^†^			Water stressed^†^		
	Soil available N (kg/ha)	Shoot N (%)	Root N (%)	Soil available N (kg/ha)	Shoot N (%)	Root N (%)
Pre-sown soil	49.93 ± 2.67^c^	—	—	49.93 ± 2.67^c^	—	—
Control	83.63 ± 2.65^b^	1.41 ± 0.13^e^	1.45 ± 0.14 ^d^	77.35 ± 2.09^b^	1.21 ± 0.06 ^b^	1.40 ± 0.04^b^
*B. liaoningense* strain D (Bl-D)	87.81 ± 4.58^b^	1.63 ± 0.12^de^	1.65 ± 0.02 ^cd^	79.47 ± 2.64^b^	1.16 ± 0.06 ^b^	1.56 ± 0.01 ^b^
*B. japonicum* strain I (Bj-D)	87.81 ± 3.24^b^	1.83 ± 0.13^cd^	1.78 ± 0.05^c^	81.56 ± 4.28^b^	1.39 ± 0.17^b^	1.67 ± 0.03^b^
*B. japonicum* strain K (Bj-K)	85.66 ± 3.48^b^	1.50 ± 0.05^de^	1.54 ± 0.06^cd^	77.35 ± 2.09^b^	1.33 ± 0.06^b^	1.46 ± 0.06^b^
*B. japonicum* strain M (Bj-M)	85.72 ± 3.86^b^	1.46 ± 0.09^e^	1.68 ± 0.11^cd^	79.47 ± 4.18^b^	1.35 ± 0.12^b^	1.68 ± 0.06^b^
*B. elkanii* strain USDA-61 (Be-61)	112.90 ± 3.24^a^	2.2 ± 0.13^b^	2.36 ± 0.13^ab^	104.53 ± 2.65^a^	2.15 ± 0.02^ab^	2.45 ± 0.09^a^
*B. elkanii* strain USDA-94 (Be-94)	110.88 ± 3.91^a^	2.11 ± 0.09^bc^	2.10 ± 0.09^b^	102.44 ± 2.09^a^	1.91 ± 0.12^b^	2.35 ± 0.17^a^
*B. diazoefficiens* strain USDA-110 (Bd-110)	114.99 ± 3.86^a^	2.50 ± 0.10^a^	2.56 ± 0.13^a^	108.71 ± 4.18^a^	2.47 ± 0.23^a^	2.49 ± 0.15^a^

^†^Values are the means of six-replication ± standard error. Values with the same superscripts within column indicate no significant difference at *P* ≥ 0.05.

Correspondingly, increased available nitrogen in the soybean rhizosphere also improved plant nitrogen status. The total nitrogen content in the shoots of soybean plants significantly increased due to the inoculation of Rtx-producing bradyrhizobial strains under watered conditions. However, the inoculation of one of the Rtx-producing strains, *B. elkanii* USDA-94, was statistically on par with the inoculation of another strain, *B. elkanii* USDA-61, and the results of both of these were significantly different from those of the inoculations of other non-Rtx-producing strains under water-stressed conditions (Table 3). The maximum increase in total nitrogen content was found in the shoots of soybean plants inoculated with *B. diazoefficiens* strain USDA-110 under watered (2.50 ± 0.10%) and water-stressed (2.47 ± 0.23%) conditions. The total nitrogen content of the roots was higher than that of the shoots of soybean grown under both watered and water-stressed conditions (Table 3). The total nitrogen content in the roots of soybean plants was positively influenced by all Rtx-producing strains and was significantly superior to that of treatments with inoculations of non-Rtx strains. The shoot nitrogen content was also found to be maximum in plants inoculated with *B. diazoefficiens* USDA-110 under watered (2.50 ± 0.1%) and water-stressed (2.49 ± 0.15%) conditions, which was followed by that of plants inoculated with Rtx-producing strains.

### Principal component analysis and cluster analysis on the overall impact of bradyrhizobial strain (rtx and non-rtx) inoculation of soybean under water-stressed conditions

**Figure 4:**
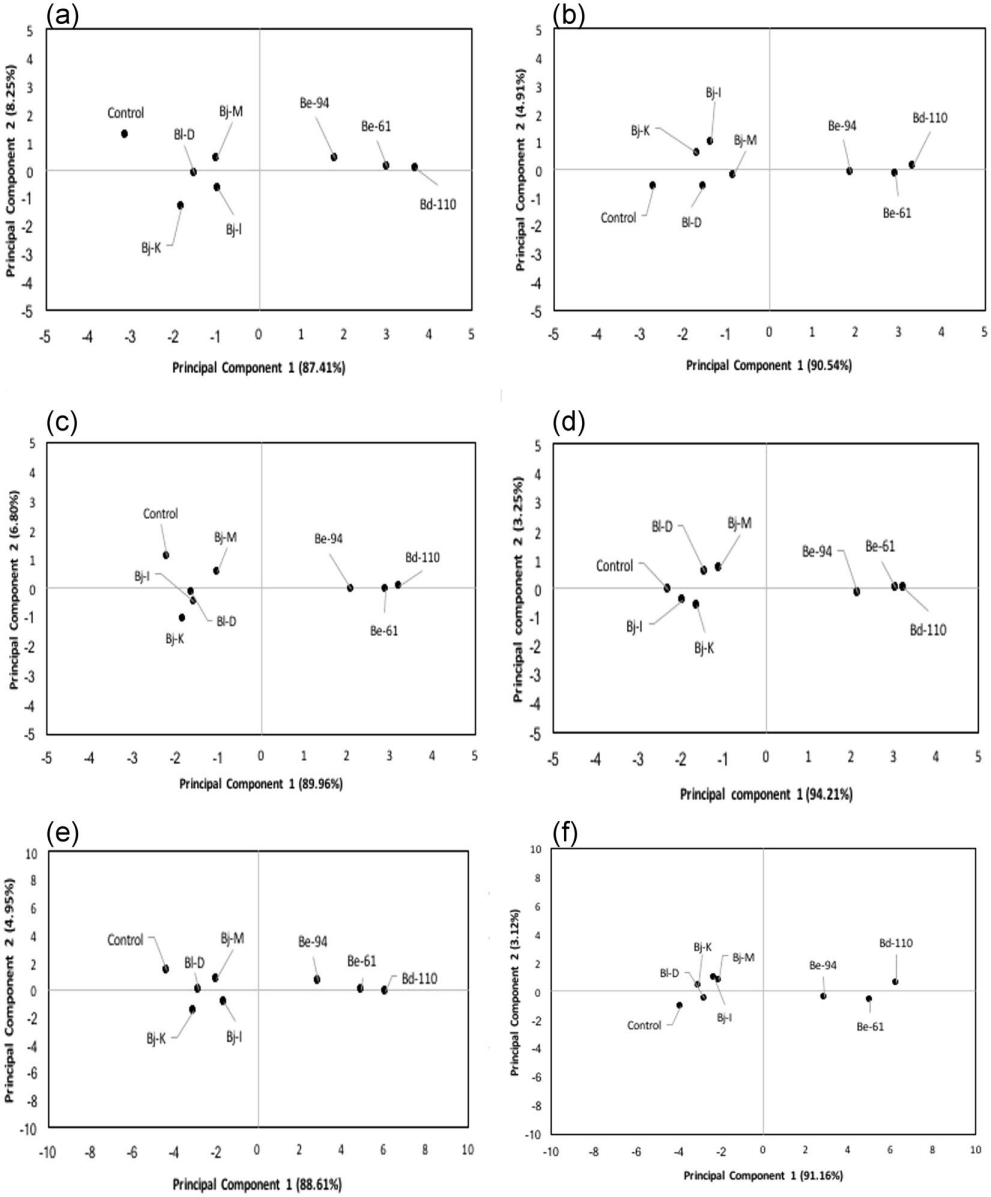
Principal component analysis (PCA) showing the inoculation effects of Rtx and non-Rtx bradyrhizobial strains for the image-based analysis of canopy measurements *vs* selected rhizosphere and plant growth parameters (**a**,**b**) specific to root nodulation and N fixation; canopy measurements *vs* rhizosphere soil parameters (**c**,**d**) related to microbial activities and plant nutrition; and canopy measurements *vs p*lant growth parameters (**e**,**f**) related to physiological and yield traits in soybean plants under watered and water-stressed conditions.

**Figure 5:**
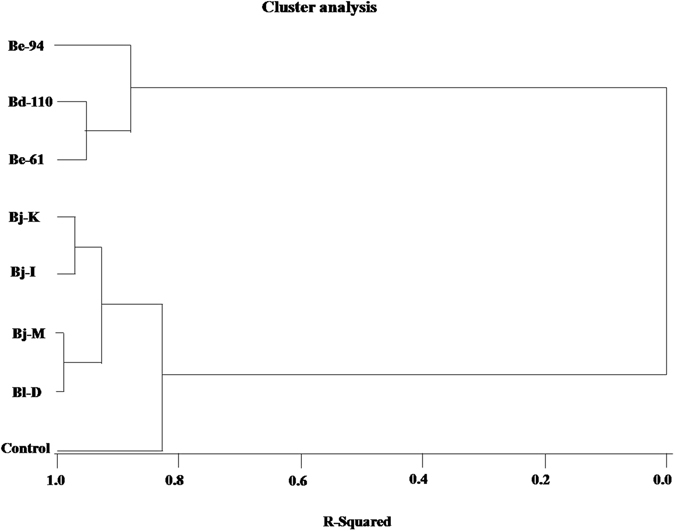
Multivariate analysis based on Ward’s minimum variance cluster analysis showing the effects of inoculation of Rtx and non-Rtx bradyrhizobial strains on plant rhizosphere and growth parameters in soybean plants under combined watered and water-stressed conditions using the CLUSTER procedure and the software package SAS^®^ 9.3 (SAS Institute, USA).

The principal component analysis showed that both the principal component 1 (PC1) and component 2 (PC2) for image pixel-based phenomics data with plant growth, soil, nodulation and N-fixation parameters exhibited great variability (Fig. 4a–f). Under watered, water-stressed and combined conditions, the PCA represented 95.66, 95.45 and 94.67% of the variability of select nodulation and N-fixation parameters; 96.76, 97.46 and 96.26% of the variability of soil parameters; and 93.56%, 94.44 and 93.39% forplant growth parameters, respectively (Fig. 4a–f & Supplementary Fig. S2). The biplot chart clearly shows that the control (no-inoculation) and non-Rtx strain inoculations share common characteristics of low impact on soybean compared to higher impacts by Rtx strains *B. elkanii* USDA-94 and USDA-61 (Be-61 and Be-94) and *B*. *diazoefficiens* strain USDA-110 (Bd-110) under water-stressed conditions. The multivariate analysis clustering based on Ward’s minimum variance analysis showed two major and distinct clusters (with a root mean square distance of 9.798 between observations) between the Rtx strains of *B. elkanii* USDA-94 and USDA-61 (Be-61 and Be-94) inoculation [together with the strain *B*. *diazoefficiens* USDA-110 (Bd-110)] treatments and non-Rtx strain inoculation treatments, including uninoculated controls; these results therefore indicate comparable characteristics between these two groups of strains and suggest their similarities within clusters (Fig. 5 & Supplementary Table ST1).

### Correlation of parameters obtained from direct quantification and RGB based analysis

Correlation analysis showed direct relationship between variables of RGB image based analysis and direct quantification from soybean- bradyrhizobial treatments under watered and water stressed conditions (Supplementary Table ST2). The correlation co-efficient (r-value) between the number of nodules *vs* blue pixels and number of nodules *vs* green pixels was estimated to be 0.677 and 0.775, respectively under watered conditions. The same was estimated to be 0.794 and 0.861 (which was supported by significant p-value) under water stressed conditions, indicating the plausibility of assessing the nodulation by examining plant surface phenotypes or canopy features like cool canopy and high canopy greenness (Table 4). Like nodulation efficiency, variables of RGB image based measurements have also been found to be directly correlated with the root, shoot nitrogen and total chlorophyll content in both watered and water stressed conditions. Also, the secondary factors were correlated more with green pixels than blue pixels and under water stressed conditions. Correlation analysis of watered and stressed plants were performed independently, and the results reveal that the r-values between the samples were not significantly different as indicated by p-values (Table 4).

**Table 4:** Comparison of correlation co-efficient (r-values) based on correlation analysis of variables from destructive (direct quantification) and non-destructive (RGB analysis) assay methods in watered and stressed plants.

Variable	Variable wise	r-value (watered conditions)	P value	r-value (water-stressed conditions)	P value	Comparison of r values between ‘watered’ and ‘water stressed’ conditions	
						Z- score	P value
No. of nodules	Blue pixels	0.677	0.065	0.794	0.019	−0.409	0.341
No. of nodules	Green pixels	0.775	0.024	0.861	0.006	−0.415	0.339
Root N	Blue pixels	0.754	0.031	0.895	0.003	−0.734	0.232
Root N	Green pixels	0.888	0.003	0.927	0.001	−0.361	0.351
Shoot N	Blue pixels	0.665	0.072	0.831	0.011	−0.614	0.270
Shoot N	Green pixels	0.848	0.008	0.897	0.003	−0.331	0.370
Chlorophyll (a + b)	Blue pixels	0.779	0.023	0.858	0.006	−0.387	0.349
Chlorophyll (a + b)	Green pixels	0.846	0.008	0.921	0.001	−0.566	0.286

## Discussion

Legume-rhizobia symbiosis is an important source of nitrogen for both natural and agricultural ecosystems. It is an appropriate model for understanding the mechanisms responsible for the evolutionary persistence of cooperation, despite conflicts of interest among symbiotic partners^25^. Abiotic stresses are known to induce ethylene biosynthesis, which inhibits overall plant growth and affects legume-*Rhizobium* symbiosis. To counter the negative effects of ethylene on nodulation, the rhizobial partners of the symbiotic system employ at least two strategies: the production of the rhizobitoxine (Rtx) protein and the ACC deaminase enzyme, which reduce the amount of ethylene synthesized by host legumes. Rhizobitoxine production by *Bradyrhizobium elkanii* is known to suppress ethylene biosynthesis in the host plant *Macroptilium atropurpureum* and enhance nodulation through rhizobitoxine-mediated inhibition of one of the key enzymes in the ethylene biosynthetic pathway^10–13^. In the present study, PCR-based screening of the *rtxA* gene resulted in expected amplification (~2500-bp product) only in the *Bradyrhizobium elkanii* strains USDA-61 and USDA-94 and not in *B. diazoefficiens* USDA-110. However, the use of degenerate primers in PCR resulted in amplification (~1250-bp product) in all three bradyrhizobial USDA strains. In contrast, all 83 native rhizobial and commercial bradyrhizobial strains failed to show *rtxA* gene amplification in the PCR screening using degenerate primers. Although rhizobitoxine production was not quantified, PCR screening for the *rtxA* gene (encoding rhizobitoxine protein subunit-A) confirmed the Rtx-producing trait in the bradyrhizobial USDA strains. Ruan *et al*.^26^ isolated the *rtxA* gene (formally both *rtxA*and *rtxB*) from *B. elkanii* USDA-61 and reported that *rtxA* mutants do not accumulate serinol in nodules and do not produce rhizobitoxine in culture or nodules. It was also demonstrated that at least the *rtxA* and *rtxC* genes are responsible for rhizobitoxine biosynthesis in free-living *B. elkanii* based on mutagenesis experiments and the quantification of rhizobitoxine intermediates in culture using LC/MS^13, 27, 28^. In *B. japonicum*, most genes involved in nodulation and symbiotic nitrogen fixation are clustered in an approximately 410-kb region of an 8.7-Mb chromosome^29, 30^. The *rtxA* and *rtxC* genes of *B. elkanii* are located in a genomic region that also harbours nodulation and symbiotic nitrogen fixation genes. Interestingly, the *rtx* cluster and upstream *noeE* gene are almost conserved in the efficient-symbiotic strain *B. japonicum* USDA-110^30^. Non-amplification of the *rtxA* gene from *B. japonicum* USDA-110 (recently named *B. diazoefficiens* USDA-110 by Delamuta *et al*.^31^ in our PCR screening using *B. elkanii*-specific primers could be due to the fragmentation of the C-terminal region of the *rtxA* gene; hence, its expression requires a translational shift^30^. Therefore, it is generally accepted that rhizobial genes involved in legume-rhizobia symbiosis have evolved by horizontal gene transfer and genomic rearrangements. Recently, the comparative analyses of genomic sequences of *B. elkanii*, *B. japonicum* and *Xanthomonas oryzae* have suggested that the *rtx* genes of the cluster even extend beyond the previously characterized *rtxA-C* genes via the additional genes *rtxD*, *rtxE*, *rtxF* and *rtxG* ^28, 32^.

In the present investigation, the use of a phenomics approach, which is based on RGB image analysis, clearly distinguished the canopy features of soybean inoculated with bradyrhizobial strains under both regularly watered (optimum soil moisture) and water-stressed (low soil moisture) conditions. The analysis of visible images revealed that the average number of green pixels and corresponding canopy greenness were higher in soybean plants inoculated with rhizobitoxine-producing strains than in plants inoculated with non-Rtx-producing strains and in uninoculated controls under water-stressed conditions. Visual imaging of plant foliar chlorosis has been used to identify symptoms of pathogenic strain infection and to study disease epidemiology^33^. However, visible image analysis-based canopy greenness analysis used in the present study has never been used as a selection criterion in breeding programmes for drought situations or even in normal conditions to identify plant responses among plant genotypes and genotype-microbe symbiotic interactions. A possible explanation for the improved canopy greenness in the Rtx-inoculated soybean plants is due to improved chlorophyll status, which ultimately depends on the increased bradyrhizobial nodulation and N-fixation. Hence, the results of visible image-based analysis were confirmed via the non-destructive measurements of the relative chlorophyll content of plants by the SPAD chlorophyll meter, which were highly correlated with empirically measured chlorophyll using the DMSO method^34, 35^. The inoculation of Rtx-producing *Bradyrhizobium* strains USDA-61 and USDA-94 and *B. diazoefficiens* strain USDA-110 significantly increased the relative chlorophyll content of soybean plants by 84%, 33% and 82%, respectively, compared with that of other strains and uninoculated controls under stress conditions. In most cases, inoculation with *Bradyrhizobium* USDA strains resulted in significant increases in leaf chlorophyll a, b, and a + b compared to the respective controls, both under soil moisture-stressed and non-stressed conditions. In general, Rtx-producing bradyrhizobial strains were found to be more effective than other treatments, and the Rtx-producing bradyrhizobial strains performed better in comparison with their counterparts under water-stressed or low soil moisture stress conditions.

IR thermal images of the plant canopy of watered soybean plants usually showed higher amounts of blue pixels compared to those of images of plants under water stress treatments. The IR thermal image analysis clearly established that soybean inoculation with Rtx-producing bradyrhizobial strains resulted in significantly higher amounts of blue pixels, indicating cooler canopy temperatures compared to those of plants inoculated with other non-Rtx strains and those of the plants of control treatments under water-stressed conditions. Although canopy temperature was not measured directly, IR thermal image analysis used in the present study could be used as a potential screening tool to identify general plant responses^36^ and legume-symbiotic interactions in particular while breeding drought-tolerant genotypes. Cooler canopy features of soybean plants inoculated with Rtx-producing strains could possibly be due to improved water status/low leaf water loss (LWL)/stomatal conductance via effective photosynthetic systems and N-metabolism, which ultimately depend on improved bradyrhizobial nodulation and N-fixation. Hence, IR thermal image-based analysis was reaffirmed using non-destructive measurements of photosynthetic efficiency and quantum efficiency (*Fv/Fm*) of plants. *Fv/Fm* values ranged from 0.73 to 0.78 for watered soybean plants; for moisture-stressed plants, the values were between 0.72 and 0.81. Soybean plants inoculated with bradyrhizobial strains had significantly higher *F*v/*F*m values under both watered (13% more than those of the control) and water-stressed conditions (7% more than those of the control). Leaf temperature measurements using IR thermal sensing is primarily used to study plant water relations; specifically, stomatal conductance and the rate of evaporation or transpiration from the leaf is a major determinant of leaf temperature^37–39^. A relatively low canopy temperature in water-stressed soybean plants indicates its ability to take up soil moisture and maintain a conducive water status by various constitutive or adaptive traits^36, 40, 41^. This capability, expressed at relatively low canopy temperatures, is correlated with yield under stress or other parameters of drought resistance, such as various plant growth and/or yield indices^36, 40–43^.

The effects of bradyrhizobial USDA strain inoculation on the improvement of soybean plant canopy features observed in the phenomics approach were mainly the result of enhanced rhizobial survival, competitiveness, nodule formation and N-fixation efficiency. Maximum nodulation was found in the soybean plants inoculated with Rtx strains compared to non-Rtx strains under optimum soil moisture and low soil moisture stress conditions, which emphasizes the importance of rhizobial traits on rhizobitoxine production. A positive role of rhizobitoxine in the symbiosis between *B. elkanii* USDA-61 and *Vigna radiate* has been reported^14^. Rhizobitoxine production in B. *elkanii* USDA-94 reduces ethylene evolution from the associated roots of *Macroptilium atropurpureum* and enhances nodule formation^13, 14^ and the symbiotic phenotypes of soybean^27^. The rhizobitoxine-producing bradyrhizobial strains exhibit better survival and nodulation protection in addition to conferring competitiveness to host legumes grown under abiotic stress. In the present study, plant growth and biomass accumulation were reduced when plants were grown under low soil moisture compared to optimum soil moisture conditions. However, the ill effects of moisture stress were reversed in soybean inoculated with strains of Rtx-producing *Bradyrhizobium* spp., as these strains improved plant growth parameters and resulted in the highest biomass accumulation under water-stressed conditions. *Bradyrhizobium diazoefficiens* strain USDA-110 and the Rtx strains *B. elkanii* USDA-61 and USDA-94 significantly increased the number and weight of pods in inoculated soybeans under both conditions; the inoculation of soybean plants with these cultures increased the number of pods by 141%, 138% and 132%, respectively, under moisture stress compared to those of the uninoculated controls. Rtx-producing strains showed remarkable effects on improving pod weight compared to non-Rtx strains and uninoculated controls under stressed conditions. The inoculation of Rtx strain *B. elkanii* USDA-61 had a significant effect on total seed weight under moisture stress. At the same time, there was no significant difference in 100-seed weight due to the inoculation of Rtx-producers and *B. diazoefficiens* strain USDA-110, as these treatments were superior among the bradyrhizobial strain inoculation treatments under low soil moisture stress conditions. The induction of nodulation- and other symbiosis-related genes in *Bradyrhizobium* has a positive effect on soybean growth under moderate drought stress^10, 44^. The investigation of Barbosa *et al*.^45^ confirmed that inoculation with *Bradyrhizobium* improves nitrogen assimilation, osmotic adjustment and growth parameters in *Vigna unguiculata* plants under water deficit conditions.

Improved plant growth and yield indices in soybean plants upon bradyrhizobia inoculation could be attributed to higher plant photosynthesis and nitrogen metabolism, as revealed by the better plant canopy features in the image analysis-based phenomics measurements. The observations also complement the results regarding chlorophyll content and photosynthetic efficiency. Increased plant photosynthesis and nitrogen metabolism results in the higher microbial nutritional status of the rhizosphere of soybean inoculated with Rtx strains as revealed by enhanced/improved rhizo-depositions. Microbial inoculations exert very positive roles in improving plant rhizosphere health in arid soils, and similar kinds of increased activity of microbial populations and plant physiological status have been reported in soybean plants inoculated with *Bradyrhizobium* ^23^. The PCA clearly revealed the reliability of the RGB image analysis-based phenomics approach over conventional destructive approaches in assessing symbiotic effectiveness of *Bradyrhizobium* strain inoculation in soybean under watered (87.41–89.96%) and water-stressed (90.54–94.21%) soil conditions. The MCA results showed two major and distinct clusters (with a root mean square error of 9.798) between effective (Rtx) and ineffective (non-Rtx) *Bradyrhizobium* strain inoculation treatments in soybean. The symbiotic effectiveness of both *B. diazoefficiens* strain USDA-110 and Rtx-producing bradyrhizobial strains was successfully differentiated from other ineffective non-Rtx bradyrhizobial strains through image-based phenomics approaches complemented with conventional methods in soybean grown under low soil moisture stress. Furthermore, a direct correlation between values obtained from RGB image based analysis of plant canopy features and direct measurements of plant parameters has unequivocally shown that the phenomics/phenotyping methodology described in this study is dependable alternative for screening drought tolerant genotypes in the field conditions.

Thus, phenomics techniques such as visible and IR thermal image-based RGB analysis of plant surface phenotypes or canopy features (canopy greenness and canopy temperature) can be considered as low-cost phenotyping approach and useful in assessing the effectiveness of plant-microbe interactions during edaphic stress/drought. In addition, this could also helpful to identify potential microsymbionts during large screens under similar situations. To the best of our knowledge, this is the first report on the application of plant phenomics tools, particularly the RGB-based image analysis, in assessing/trait-phenotyping plant-microbe symbiotic interactions that impart abiotic stress tolerance.

## Methods

### Bacterial cultures and growth conditions

There were 79 rhizobial isolates purified from the surface-sterilized nodules of soybean and mungbean grown in different field soils. Surface sterilization of nodules was performed in a series of washing steps under aseptic conditions as follows: washing in 70% ethanol for 1 min, 4% sodium hypochlorite for 5 min, and 90% ethanol for 30 s; 3 washings in sterile distilled water; washing in a 5% sodium thiosulfate solution for 5 min; and a final washing in sterile distilled water. A crushed nodule suspension was used for isolation and further purification of these cultures. Four commercial bradyrhizobial strains, *Bradyrhizobium liaoningense* D (Bl-D), *B. japonicum* I (Bj-I), *B. japonicum* K (Bj-K) and *B. japonicum* M (Bj-M), were used in this study. Three strains, including *B. elkanii* USDA-61 (Be-61), *B. elkanii* USDA-94 (Be-94) and *B. diazoefficiens* USDA-110 (Bd-110) (formerly called *B. japonicum* USDA-110), were obtained from the curator of the USDA-ARS, Beltsville, MD, USA. These strains were cultured in yeast extract mannitol agar medium (YEMA) containing 1% mannitol, 0.1% yeast extract, 0.02% MgSO_4_·7H_2_O, 0.05% K_2_HPO_4_ and 0.01% NaCl and few drops of congored (1/400 aqueous solution) at 28 °C.

### PCR screening of bradyrhizobial strains for rhizobitoxine production trait

All 86 strains were grown by culturing in YEMA broth at 28 °C for 48 h in a refrigerated incubator shaker (Climo-Shaker ISF1-X, Kuhner, Switzerland). Genomic DNA was extracted following the methods of both Charles and Nester (1993)^46^ and Sambrook and Russell^47^, with minor modifications. Polymerase chain reaction (PCR) amplification of the *rtxA* gene was performed using genomic DNA templates extracted from these strains in order to screen these strains for rhizobitoxine production traits. To amplify the *rtxA*gene, the primer set consisting of *rtxA* F (5′-TAG AAT TCT CCA ACG AGT GAC AGT ATG CGA-3′) and *rtxA* R (5′-CTA ACT GAA CAG CCT CAT AAC G-3′) was used^13^. Another primer set that consisted of *rtxA *F1 (5′-ACG GCT TAC GAA CTT GAT GG-3′) and *rtxA *R1 (5′-TCA GCT CGG ACA ATT GCT TA-3′) was designed for the amplification of the partial *rtxA* gene. Both of these primer sets were synthesized and procured from IDT (Integrated DNA Technologies Pvt. Ltd., Germany). The PCR mixture (25 μl) comprised 25 ng of genomic DNA, 1X polymerase buffer, each dNTP at0.2 mM, each primer at 100 nM and 1U of the *Taq* polymerase enzyme. The PCR cycle consisted of an initial denaturation at 95 °C for 5 min; 30 cycles of 95 °C for 1 min, annealing at 50 °C for 1 min and extension at 72 °C for 1 min; and a final extension at 72 °C for 6 min. PCR was performed in Biorad thermocycler 1000 (Biorad, USA), and the resulting amplicons of the *rtxA* gene were confirmed by resolving the PCR products in1.2% agarose gel after performing electrophoresis followed by observation under UV trans-illumination in a gel imaging system (SynGene, USA).

### Pot experimental setup

The pot experiment was carried out in the field of the ICAR-National Institute of Abiotic Stress Management, Baramati, India (18° 09′ 30.62”N latitude; 74° 30′ 03.08”E longitude; MSL 570 m altitude) during July–October 2013 in a completely randomized design. The mean temperature, relative humidity and rainfall during the study period was 26 °C, 73% and 104 mm, respectively. Carrier-based inocula for the four selected non-Rtx-producing, two Rtx-producing strains of *Bradyrhizobium* spp. and *B.diazoefficiens* USDA-11O were prepared. Each of the strains was cultured in 250 mL of YEMA broth at 28 °Cfor 48 h in a refrigerated incubator shaker (Climo-Shaker ISF1-X, Khuner, Switzerland), and the growth was adjusted so that1 × 10^8^–10^9^ cells/ml was equivalent to 1 OD (A600 nm = 1.0) spectrophotometrically. Each culture was then mixed with 500 g of sterile charcoal powder and air-dried under sterile conditions, which was used for both seed pelleting and soil applications. Each treatment involved 300 kg of soil mixed with 578 g of single super phosphate (SSP) and 125 g of muriate of potash (MoP). The soil was thoroughly mixed with 25 g of charcoal-based inocula on clean polythene sheets, and each pot was then filled with the 25 kg of the soil and inoculum mixture. Soybean seeds (NRC-37/Ahilya-4, a cultivar highly susceptible to low soil moisture stress) were obtained from the ICAR-Indian Institute of Soybean Research (ICAR-IISR), Indore, India. Seeds were surface-sterilized in a series of washings as follows: washing in 70% ethanol for 1 min, 4% sodium hypochlorite for 5 min, and then 90% ethanol for 30 s; washing in sterile distilled water thrice; washing in a 5% sodium thiosulfate solution for 5 min; and finally three washings in sterile distilled water. Surface-sterilized seeds were coated with 1 g of carrier-based inoculum of *Bradyrhizobium* strains and sown at a rate of 3 seeds per pot. *Bradyrhizobium* treatments divided into two sets of six replications each were subjected to regular watering and imposed water stress (low soil moisture) conditions. The water holding capacity (WHC) of the pot containing 25 kg of soil was predetermined. Control pots were provided with the required amount of water once every 5 days to maintain the soil at full water holding capacity, whereas water stress was imposed on pots by supplying half the amount of water the control pots were given. The imposition of water stress started on the 15^th^ day after sowing, and water was applied every other day after the appearance of temporary wilting points in the plants.

### Standardization of image analysis software and the visible and IR thermal imaging of the plant canopy

RGB-based image analysis software (Supplementary Fig. S3) was custom programmed/designed. The software was compatible with Windows XP and other advanced Windows versions (32 bit and/or 64 bit), and standardization was performed with colour images of defined resolution [pixels (0–10000 pixels)] for the primary colours red, green and blue and their combination/mixtures^24^. Standardized RGB analysis software was utilized for evaluating plant images obtained using visible (D90, Nikon, Japan) and infrared cameras (Vario CAM hr inspects 575, Jenoptic, Germany). Both visible and infrared cameras placed were in a fixed angle, and optical focus was standardized for simultaneous imaging of the systematically arranged pots (Supplementary Fig. S3a). Soybean canopy features were monitored using both visible and IR imaging a week before and at the time of flower initiation. From the visible and thermal images, a plant canopy area of known size (1 cm^2^) was selected and used in the image analysis by the standardized RGB analysis software^24, 36^ (Supplementary Fig. S3b).

### Measurement of relative chlorophyll content using SPAD chlorophyll meter and quantum efficiency of PSII using a chlorophyll fluorescence meter

The Minolta SPAD chlorophyll meter (SPAD-502, Minolta, Japan) was used to measure the leaf relative chlorophyll content of the treated plants. The mean of three observations from the portable chlorophyll meter was obtained from individual fully emerged 3^rd^-trifoliate leaves^48, 49^. The quantum efficiency of photosystem II was measured based on chlorophyll fluorescence during the light reaction of photosynthesis. Chlorophyll fluorescence was determined from leaf discs using a dark-acclimated Handy Plant Efficiency Analyzer chlorophyll fluorometer (FMS2, Hansatech Instruments, King’s Lynn, Norfolk, UK). The initial fluorescence (*F*0) and maximum fluorescence (*F*m) were analysed, and the quantum efficiency of open photosystem II centres–quantum yield (*F*v*/F*m) was calculated. Briefly, leaf discs were previously adapted to the dark for 30 min so that all the centres of photosystem II (PSII) were in the open state (all the primary acceptors were oxidized), and energy dissipation through heat was minimal. The *F*0 was obtained using low-intensity light (less than 0.1 mol/m^2^/s^1^), which did not induce any effect in the fluorescence variable. The *F*m was obtained using continuous light excitation (at 2500 mol/m^2^/s^1^) provided by an array of six LEDs focused on the leaf surface to provide homogeneous irradiation over a 4-mm (0.16 in)-diameter leaf surface. The fluorescence variable (*F*v) was calculated from the difference between *F*m and *F*0. The *F*v and *F*m values were used to obtain the *F*v*/F*m ratio, which indicates the quantum efficiency of PSII.

### Estimation of the chlorophyll content of soybean inoculated with bradyrhizobial strains

Fully emerged individual 3^rd^-trifoliate leaves of each plant from all treatments were collected, and the chlorophyll content was estimated in accordance with the DMSO method^50^. Leaf samples (25 mg each) were cut into fine strips/pieces and placed into a test tube containing 5 mL of DMSO. The test tubes were then incubated in darkness at 37 °C for 24 h. After extraction in the dark, a 3-mL aliquot was analysed spectrophotometrically at 649 and 665 nm (Shimadzu UV1800, Japan). Chlorophyll a, chlorophyll b and carotenoid concentrations were obtained based on the formulae proposed by Wellburn(1994)^50^.

### Measurement of nodulation, plant growth and yield parameters

Treated plants were randomly carefully uprooted, and the number of nodules was counted to measure nodulation efficiency. At harvest, plant growth parameters such as shoot length, root length, the number of branches, the total number of pods and total pod weight per plant were measured. The harvested shoot and root samples were placed into brown paper bags and kept in a hot-air oven for 7 days at 60 °C for complete desiccation, after which the dry weight of the shoots and roots were recorded. The seeds were carefully cleaned, and total seed weight per plant was recorded for all the treatments. From each replication, 100 seeds were counted randomly in triplicate, and the seed test weight per plant was determined.

### Estimation of nitrogen content of the rhizosphere soil and plant samples

Available soil nitrogen was estimated according to the potassium permanganate wet oxidation method following modifications suggested by Subbiah and Asija^51^. Two grams of rhizospheric soil was wet-digested with potassium permanganate in a micro-Kjeldahl apparatus followed by nitrogen estimation by steam distillation and chemical titration. Oven-dried plant materials (shoots and roots) from all the treatments were ground separately and used for nitrogen estimation. Total nitrogen in 1 g of plant shoots and roots along with nodule samples were analysed according to the micro-Kjeldahl apparatus method^52^. Briefly, the powdered plant samples were digested via Kjeldahl digestion after reducing nitrates with Devarda’s alloy^53^ and NH_4_^+^ and NO_3_^−^ by steam distillation.

### Statistical analysis of experimental data

Statistical analysis was carried out using the SPSS statistical software package version 16.0 (IBM SPSS, USA). The data obtained from the values of six replicate samples for each treatment were analysed by the analysis of variance (ANOVA); the treatment means were subjected to the least significant difference (LSD) at both *P* = 0.05 and *P* = 0.01, and the means were compared by Duncan’s multiple range test (DMRT). The standard error for each treatment was determined by following the standard procedures wherever required. Principal component analysis and multivariate cluster analysis for all the data obtained from six replicates of each treatment were performed to differentiate treatment effects using statistical analysis software package SAS^®^ 9.3 (SAS institute, USA). In order study the efficacy of RGB image based analysis for assessing the *Bradyrhizobium*-plant interactions, a correlation investigation was carried out between variables obtained from destructive and the non-destructive methods.

## Electronic supplementary material

Supplementary Information
