# Supplementary material for: Comparative conventional and phenomics approaches to assess symbiotic effectiveness of Bradyrhizobia strains in soybean (Glycine max L. Merrill) to drought
Source: Sci Rep. 2017 Jul 31;7:6958. doi: 10.1038/s41598-017-06441-3 (PMC5537308; doi:10.1038/s41598-017-06441-3)
Supplement: Supplementary file 1 — Supplementary Information [file 41598_2017_6441_MOESM1_ESM.pdf]

Comparative conventional and phenomics approaches to assess symbiotic effectiveness of *Bradyrhizobia* strains in soybean (*Glycine max* L. Merrill) to drought

Venkadasamy Govindasamy[1, 2*$], Priya George[1,$], Lalitkumar Aher[1#], Shunmugiah V. Ramesh[3#], Arunachalam Thangasamy[4#], Sivalingam Anandan[4#], Susheel Kumar Raina[1,5&,] Mahesh Kumar[1&], Jagadish Rane[1$], Kannepalli Annapurna[2&] and Paramjit Singh Minhas[1&]

Supplementary Table ST1: Multivariate cluster analysis based on Ward's Minimum Variance Cluster Analysis for the cluster history showing effect of inoculation of Rtx and non-Rtx bradyrhizobial strains on plant rhizosphere and plant growth parameters in soybean under combined watered and water stressed conditions with the CLUSTER procedure adapted using The SAS system software package SAS® 9.3 (SAS institute, USA).

Cluster History						
**Root-Mean-Square Distance between observations**					9.797959	
**Number of Clusters**	**Clusters Joined**		**Frequency**	**Semi-partial R-Square**	**R-Square**	**Tie**
**7**	BI-D	Bj-M	2	0.0118	0.988	-
**6**	Bj-I	Bj-K	2	0.0171	0.971	-
**5**	Be-61	Bd-110	2	0.0195	0.952	-
**4**	CL7	CL6	4	0.0253	0.926	-
**3**	CL5	Be-94	3	0.0474	0.879	-
**2**	Control	CL4	5	0.0526	0.826	-
**1**	CL2	CL3	8	0.8263	0.000	-

Supplementary Table ST2:

Correlation analysis between variables obtained from destructive (direct quantification) and non-destructive (RGB analysis) assay methods obtained from soybean-bradyrhizobial treatments under watered and water stressed conditions.

Variable	Watered†						Water stressed†					
	Blue pixels	Green pixels	No. of nodules	Root N	Shoot N	Chlorophyll (a+b)	Blue pixels	Green pixels	No. of nodules	Root N	Shoot N	Chlorophyll (a+b)
**Blue pixels**	1	0.625	0.775	0.888	0.848	0.846	1	0.868	0.861	0.927	0.897	0.921
**Green pixels**	0.625	1	0.677	0.754	0.665	0.779	0.868	1	0.794	0.895	0.831	0.858
**No. of nodules**	0.775	0.677	1	0.954	0.908	0.787	0.861	0.794	1	0.926	0.977	0.951
**Root N**	0.888	0.754	0.954	1	0.976	0.915	0.927	0.895	0.926	1	0.958	0.964
**Shoot N**	0.848	0.665	0.908	0.976	1	0.916	0.897	0.831	0.977	0.958	1	0.993
**Chlorophyll (a+b)**	0.846	0.779	0.787	0.915	0.916	1	0.921	0.858	0.951	0.964	0.993	1

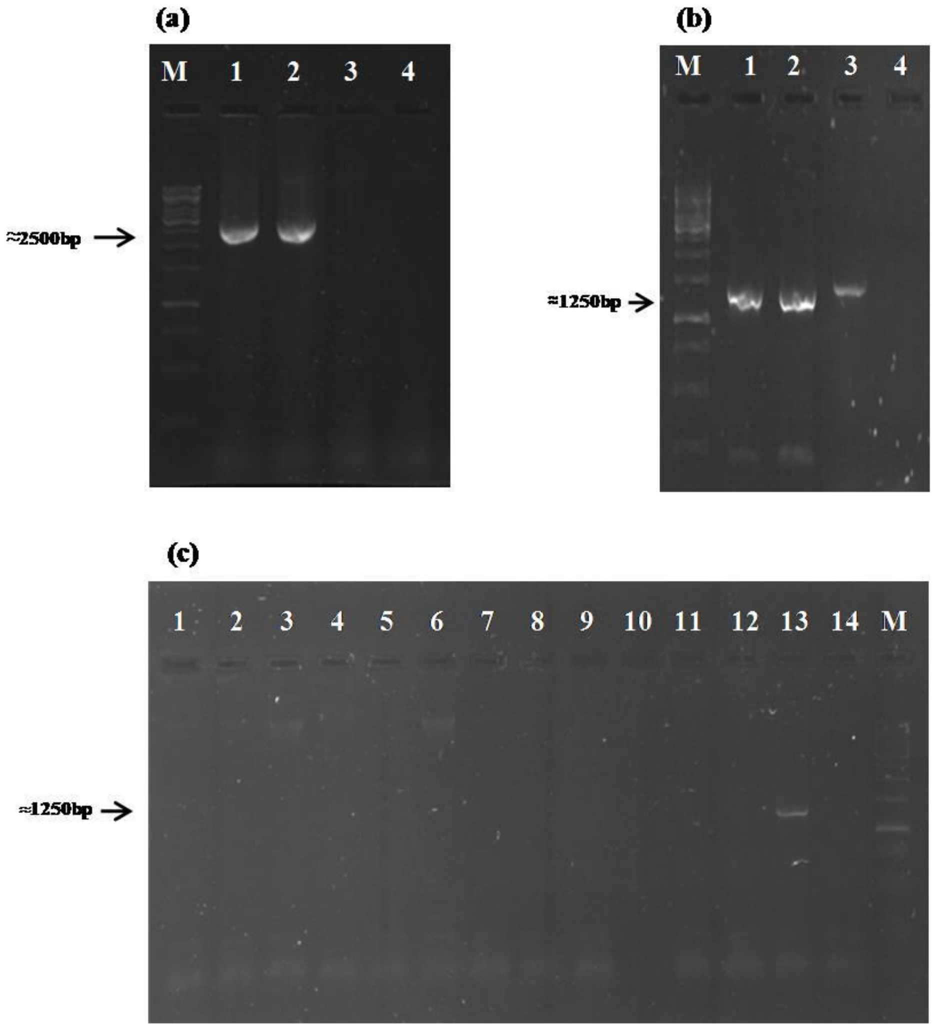

Supplementary Fig. S1 PCR amplification of *rtxA* gene from *Bradyrhizobium* USDA strains using (a) published primer set[25] (Lane M: 1000bp Marker; Lane 1: Be-61; Lane 2: Be-94; Lane 3: Bd-110; Lane 4: Negative control), (b) designed degenerate primer set (Lane M: 1000bp Marker; Lane 1: Be-61; Lane 2: Be-94; Lane 3: Bd-110; Lane 4: Negative control) and (c) representative gel image of PCR based screening of native or non-Rtx strains for *rtxA* gene amplification using degenerate primer set (Lane 1-12: Native bradyrhizobial strains; Lane 13: Bd-110; Lane 14: Negative control; Lane M: 1000bp Marker)

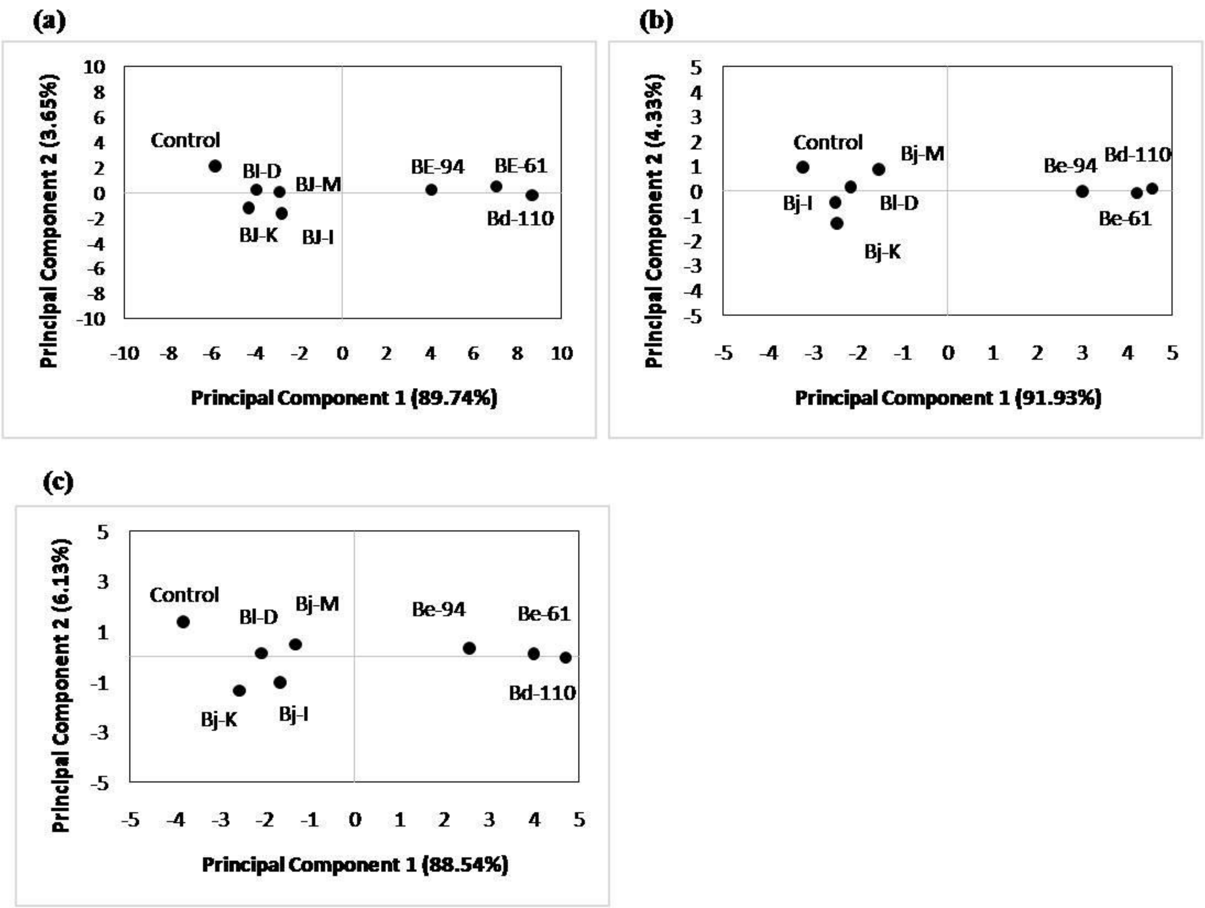

Supplementary Fig. S2 Principal component analysis (PCA) showing the inoculation effect of Rtx and non-Rtx bradyrhizobial stains for the image based analysis of canopy measurements *vs* selected rhizosphere and plant growth parameters (a) specific to root nodulation and N-fixation; canopy measurements *vs* rhizosphere soil parameters (b) related to microbial activities and plant nutrition; canopy measurements *vs* plant growth parameters (c) related to physiological and yield traits in soybean under combined watered and water stressed conditions.

(a)

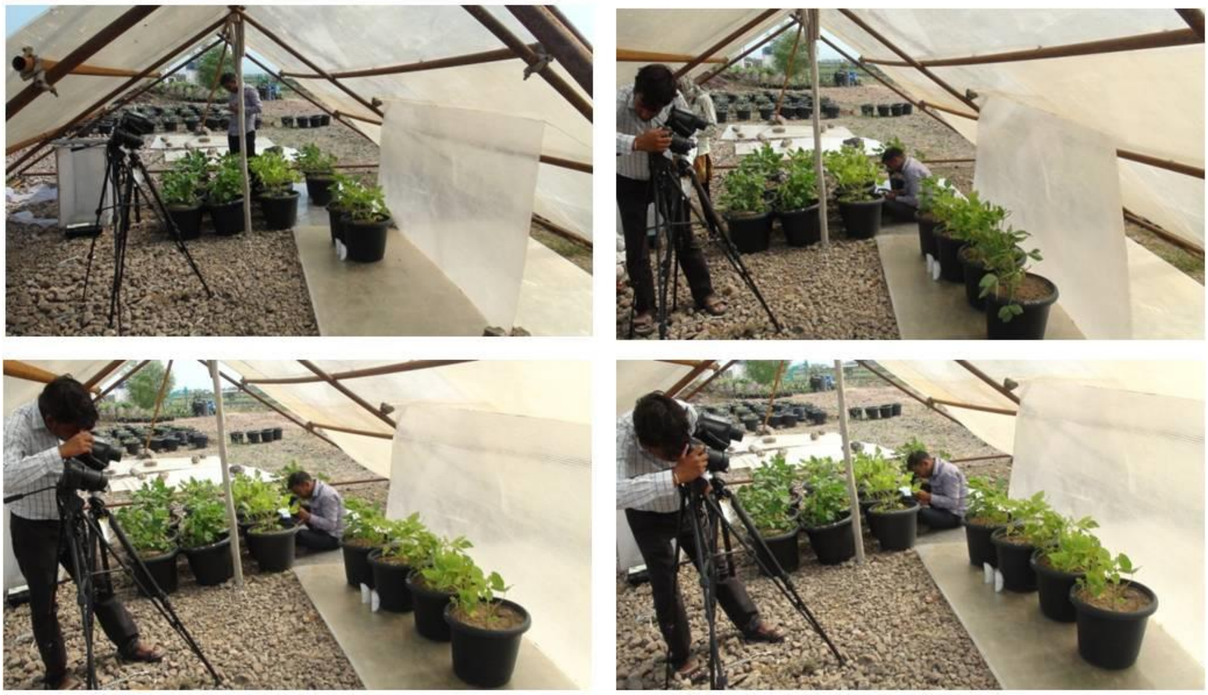

(b)

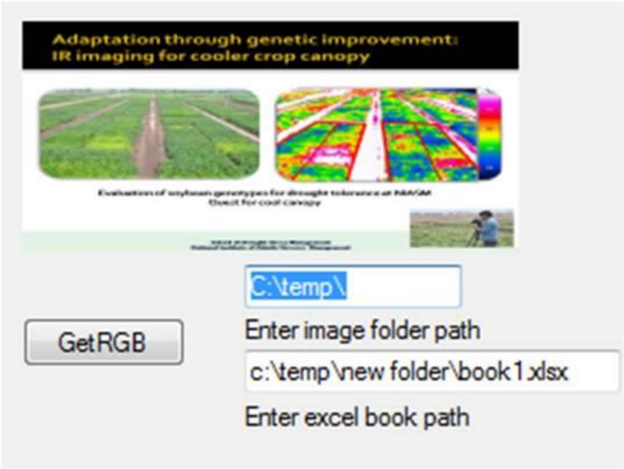

Supplementary Fig. S3 Representative pictures showing methodology adapted in visible and IR imaging process (a) and view of indigenously designed/ programmed RGB analysis software (b) used in the plant canopy image analysis.
